# Myeloid-Derived Suppressor Cells in Immune Microenvironment Promote Progression of Esophagogastric Junction Adenocarcinoma

**DOI:** 10.3389/fonc.2021.640080

**Published:** 2021-03-29

**Authors:** Ying Wang, Haiyan Sun, Ningning Zhu, Xianxian Wu, Zhilin Sui, Lei Gong, Zhentao Yu

**Affiliations:** ^1^ Department of Esophageal Cancer, Tianjin Medical University Cancer Institute and Hospital, National Clinical Research Center for Cancer, Key Laboratory of Cancer Prevention and Therapy, Tianjin’s Clinical Research Center for Cancer, Tianjin, China; ^2^ Department of Thoracic Surgery, Shenzhen Center, Cancer Hospital Chinese Academy of Medical Sciences, Shenzhen, China

**Keywords:** adenocarcinoma of the esophagogastric junction, tumor microenvironment, immune cells, myeloid-derived suppressor cells, immunological suppression

## Abstract

Adenocarcinoma of the esophagogastric junction (AEG) is a fatal disease. Accumulating evidence indicates that, for a comprehensive understanding of AEG, studies should be conducted not only to investigate tumor cells, but also the tumor microenvironment (TME). In this study, we collected AEG patient data from The Cancer Genome Atlas, and used the CIBERSORT algorithm to analyze tumor-infiltrating immune cell profiles. The levels of CD8^+^ T cells and M0 and M2 macrophages were relatively high in AEG tissues. M2 macrophages were abundant in G3 tumors, and neutrophils were associated with poor prognosis. Myeloid-derived suppressor cells (MDSCs) represent a heterogeneous population of immunosuppressive cells which share a similar origin to neutrophils and macrophages. We further analyzed the levels of MDSCs in AEG patients and healthy donors (HD) using flow cytometry. MDSC levels were elevated at tumor sites, with polymorphonuclear MDSCs (PMN-MDSCs) being the predominant subtype. Circulating MDSCs partly represented cells at the tumor site. We observed that PMN-MDSC levels at tumor sites were positively correlated with advanced staging, low grade, lymph node metastasis, and HER2^−^ status. Immunohistochemistry and immunofluorescence analyses indicated that activation of the STAT3 and NF-κB pathways in MDSCs may be a potential mechanism for cancer progression. Our studies provided a comprehensive perspective involving tumor-infiltrating immune cells, and detailed insights into the proportion of MDSCs in AEG and their clinical significance. Together, these findings may improve our current understanding of cancer progression involving tumor-infiltrating immune cells in the TME.

## Introduction

The esophagogastric junction (EGJ) is a special anatomical position that exhibits a high risk of adenocarcinoma development (1). In recent years, AEG has garnered considerable attention owing to its increasing incidence. Surgical resection is currently the most effective treatment for AEG. Despite advances in immunotherapy and targeted treatments, certain patients with AEG continue to demonstrate poor responses to existing treatments (2, 3). In the present era, personalized medicine has motivated us to understand and explore the underlying mechanisms involved in chemotherapeutic drug resistance.

Some studies have indicated that complex interactions involving tumor, immune, and stromal cells result in characteristics that promote cancer progression, angiogenesis, cellular invasion, and metastasis (4, 5). Tumor-infiltrating immune cells migrate from peripheral tissues to tumor sites and play important roles in anti- and pro-tumor activities (6). CD8^+^ cytotoxic T lymphocytes (CTLs) are key immune cells that eliminate cancer cells in concert with the major histocompatibility complex class I (MHC-I) molecules (7). During tumor progression, CTLs undergo dysfunction and exhaustion, leading to the occurrence of immune-related tolerance and immunosuppression in the TME (8). CTLs engage in the establishment of positive crosstalk with certain immune cells, including CD4^+^ T cells, NK cells, M1 macrophages, and dendritic cells (DCs), while result in the establishment of negative crosstalk with immunosuppressive cells, such as cancer-associated fibroblasts (CAFs), MDSCs, M2 macrophages, regulatory T cells (Tregs), and cancer cells (9, 10). Owing to the heterogeneity of tumors, there are differences in the number and distribution of immune cells in different patients at different pathological stages (11). Accordingly, to accurately identify immune cell infiltration landscapes in AEG patients, a large repository of transcriptome data from The Cancer Genome Atlas (TCGA) can be utilized. The CIBERSORT algorithm can be used to analyze 22 different types of immune cell infiltration in the TME. CIBERSORT has been successfully validated in breast, lung, and liver cancers (12).

MDSCs represent a heterogeneous population of immunosuppressive cells that are undergo arrest at different stages of differentiation and mainly infiltrate tumor lesions (13, 14). Accumulating evidence indicates that PMN-MDSCs and monocytic myeloid-derived suppressor cells (M-MDSCs) are generated from the normal progenitors of neutrophils and monocytes, respectively, with subsequent conversion to MDSCs; however, the biological factors responsible for such conversion have not been identified thus far (15). Therefore, PMN-MDSCs can be regarded as pathologically activated neutrophils in patients with cancer and those with other diseases, and may play important roles in regulating immune responses (16). Some evidences indicated that monocytes/M-MDSCs undergo differentiation into tumor-associated macrophages (TAMs) after migrating to tumor sites as evidenced in models of pancreatic adenocarcinoma, mammary tumors, and several other disorders (15). The term TAMs is used to define macrophages with the M2 phenotype; TAMs exert anti-inflammatory and pro-tumoral effects, similar to M-MDSCs, and directly affect multiple steps of tumor development, such as cancer cell proliferation, stemness, invasiveness, angiogenesis, and immunosuppression (17). Owing to the heterogeneity observed in both morphology and function and the absence of specific cell-surface markers, MDSCs are not included in the above-mentioned 22 immune cell types. Some studies have shown that increased levels of peripheral blood MDSCs correlate with clinical staging, overall survival, vasculogenesis, metastatic burden, and tumor evasion involving several types of tumors. The same trend is also observed in MDSCs infiltrating tumors (13). Stage III or IV gastric cancer patients present with more abundant MDSCs than stage I and II gastric cancer patients, and those with higher levels of MDSCs exhibit poorer prognoses (18). Other studies have shown that increased MDSC levels in patients with colorectal cancer and cervical carcinoma are correlated with cancer stage and metastasis (19, 20). Elevated numbers of M-MDSCs are associated with known negative prognostic markers in prostate cancer patients, including high levels of lactate dehydrogenase and prostate-specific antigen (21). Therefore, circulating MDSCs are considered as potential markers for tumor progression in cancer immunotherapy, and are an important early indicator for predicting clinical responses to chemotherapy.

MDSCs lead to the occurrence of metastasis through both immunological and non-immunological mechanisms (22). On one hand, immunological mechanisms include induction of immunosuppressive cells, production of reactive oxygen and nitrogen species, depletion of metabolites critical for T cell functions, blockade of lymphocyte homing, expression of negative immune checkpoint molecules, and expression of ectoenzymes that regulate adenosine metabolism (23, 24). On the other hand, non-immunological mechanisms include degradation of the extracellular matrix, promotion of tumor cell invasion, enhanced angiogenesis, and participation in the formation of pre-metastatic niches (25, 26). The Janus kinase (JAK)-STAT signaling pathway is usually observed at sites of cancer metastasis. Many chemokines, cytokines, and growth factor receptors are activated by their cognate ligands, leading to JAK recruitment and stimulation and activation of STAT proteins (27, 28). In the context of MDSC activation, STAT1 and STAT3 are the main contributors to the immunosuppressive mechanisms involved (29).

The present study revealed the immune cell infiltration landscape of AEG tumor patients, focusing on the potential relationship between MDSCs and clinicopathological characteristics. A possible regulatory mechanism involving the STAT3 and NF-κB signaling pathway has been presented as well.

## Materials and Methods

### Data From the TCGA Cohort

The transcriptome expression profiles and corresponding clinical information of AEG patients were drawn from 194 samples (174 tumor samples *vs.* 20 normal control samples) in the TCGA. These samples were obtained from esophageal adenocarcinomas patients and stomach adenocarcinoma patients. The expression profile of each sample and corresponding clinical dataset was logically organized.

### Evaluation of Tumor-Infiltrating Immune Cells

Tumor infiltrating immune cells were calculated by using the CIBERSORT algorithm. CIBERSORT is an analytical tool, based on gene expression that was reported to predict the fractions of multiple immune cells types in the gene expression profiles. That is to say, CIBERSORT transformed the expression of genes into the levels of immune cells through analyzing the compositions and proportions of tumor-infiltrating immune cells in tissue samples. A p-value was also derived for the deconvolution of each sample. Using the filtered data, the fractions of immune cells in each AEG sample were displayed in the form of a bar plot, corheatmap, and heatmap.

### Correlation Analysis Between Tumor-Infiltrating Immune Cells and Clinical Information

Only samples with immune cell files in the TCGA datasets meet the filter conditions were selected for the subsequent clinical analysis. The filtered immune cell expression matrix was combined with the clinical information matrix from TCGA. Five-year overall survival of the filtered immune cells and the correlations between the cells and clinical information of AEG were evaluated.

### Patients and HD

In this study, clinical samples were collected from 46 AEG patients who received radical esophagectomy at the Department of Esophageal Oncology of Tianjin Medical University Cancer Institute and Hospital from January 2018 to May 2020. We collected fresh AEG tumor tissues (TT) and corresponding adjacent normal tissues adjacent (AT, >5 cm from the lesion) from 46 patients. TT and AT samples were confirmed by using hematoxylin and eosin (H&E) staining. Patient blood (PB) samples from 27 AEG patients. Then 2 ml peripheral blood was collected from HD (n = 7). This project was approved by the Ethics Committee of Tianjin Medical University (bc2020097). All experiments involving humans were performed in accordance with the principles of Declaration of Helsinki. Written consents were obtained from each patient and healthy donor.

#### Isolation of MDSCs and Flow Cytometry Analysis

Twenty-seven venous PB samples were collected aseptically before the surgical procedure. Plasma samples were collected by centrifugation (1,500 rpm/10 min) and the supernatants were stored at −80C for later analysis. Mononuclear cells were isolated by using RBC Lysis Buffer (Solarbio). Forty-six cases of AEG tumor tissues and adjacent normal tissues were obtained immediately after surgery. For isolation of single-cell suspension, fresh tissue samples were minced into small pieces then they were dissociated by using the enzyme digestion method. The resulting cell suspension was filtered through 70-mm mesh filter (Biosharp). To determine the frequency and phenotype of MDSCs, multicolor FACS analysis was done by using the following Abs (Biolegend): anti-CD45/APC-Cy7, anti-HLA-DR/FITC, anti-CD11b/APC, anti-CD33/PE, anti-CD14/Pe-Cy7, anti-CD15/PerCP-Cy5.5, and their respective isotype controls. Viable cells were selected by gating using forward and side scatter characteristics. All samples were analyzed using a FACSAria flow cytometer (Becton Dickinson, Mountain View, CA) and at least 50,000 events were acquired for each analysis. Data were analyzed using FlowJo software. Results are expressed as the percentage of positive cells.

### Immunohistochemical Validation

AEG tissues were formalin-fixed and paraffin-embedded for immunohistochemical staining. Tissue sections were incubated with rabbit p-STAT3(Cell Signaling#9145) mAbs, and mouse CD33 (ZM-0045) mAbs overnight at 4˚C, followed by goat anti-mouse or anti-rabbit IgG secondary Abs conjugated with streptavidin-HRP (ZSGB-Bio, kit5010). Tissue sections were developed using a diaminobenzidine staining kit (ZSGB-Bio, Beijing, China) and observed using an Olympus BX51 microscope (Olympus, Tokyo, Japan). Mouse/rabbit isotype IgG1 were used as the negative controls.

### Isolation of MDSCs and Double Immunofluorescence Show Co-Localization

Single-cell suspensions of tumor tissues were obtained as described above. Then, CD15^+^cells were isolated by using the magnetic-activated cell sorting (MACS) system with CD15 magnetic beads (130-093-634; Milteny Biotec) and MS column (130-042-201; Milteny Biotec). CD15^+^Cells were spun onto slides using a Cytospin 4 centrifuge (Shandon, UK) at 1,000 rpm for 3 min. CD15^+^cells were fixed in 4% paraformaldehyde 10 min and were permeabilized with 0.2% Triton X-100 for 10 min at room temperature. After blocking with normal non-immune goat serum for 30 min, cytocentrifuge smears of cells were incubated with mouse CD33(ab30371; 1:100 dilution) and rabbit p-STAT3 (Cell Signaling#12640; 1:100 dilution) or NF-κB or iNOS or ARG antibodies. Then, the cytocentrifuge smears of cells were stained with RBITC-conjugated anti-mouse IgG and FITC-conjugated anti-rabbit IgG antibodies, nuclei were stained with 4′,6-dia midino-2-phenylindole (DAPI). Negative control staining was performed by omission of the primary antibody. Immunofluorescence images were observed by using a ZEISS fluorescence microscope (ZEISS,Axio Imager2).

### Triple Immunofluorescence Analysis

The slides were microwaved in EDTA buffer (Opal-7 Color Manual IHC kit; PerkinElmer, Waltham, MA, USA) and cooled for 1 h. Sections were then incubated in Antibody Diluent/Blocking Buffer (Opal-7 Color Manual IHC kit; PerkinElmer) for 10 min at room temperature and then incubated with primary antibodies. Triple staining was performed with CD8/FOXP3/CD33 overnight at 4°C. Samples were washed three times in TBST for 3 min each and then incubated in Polymer HRP (Ms + Rb) (Opal-7 Color Manual IHC kit; PerkinElmer). Samples were rinsed three times in TBST for 3 min each and then incubated in Opal Fluorosphore working solution (Opal-7 Color Manual IHC kit; PerkinElmer) for 10 min at room temperature. Samples were rinsed in TBST, microwaved in EDTA buffer, and then used DAPI Counterstain (Opal-7 Color Manual IHC kit; PerkinElmer) to visualize nuclei. In triple staining, CD33 were labeled with Opal 520 Fluorosphore (Green), CD8 was labeled with Opal 570 Fluorosphore (Red), and FOXP3 was labeled with Opal 690 Fluorosphore (white). For taking photomicrographs we used ZEISS fluorescence microscope (ZEISS, Axio Imager2). The ratio of positive cells in immune cells was defined as the ratio of the number of each positive immune cells to the whole number of each immune cells (red, white, and green) in a 4-mm^2^ field of view, from five separate areas.

### ELISA Detect Cytokine Levels and Arginase Activity Assay

Plasma samples were collected from HD and AEG patients. The levels of various cytokines, including and IDO were analyzed using ELISA kits (Dakewe Biotech; TGF-ß1/IL6 analysis; Jiangsu meibiao biology; analysis IDO) following the manufacturer’s instructions. Arginase1(ARG1) activity was assessed with Arginase Assay Kit (Jiangsu meibiao biology) using the UV-VIS Spectrophotometer (BECKMAN DU-800, USA).

### Statistical Analysis

**Figure 1:**
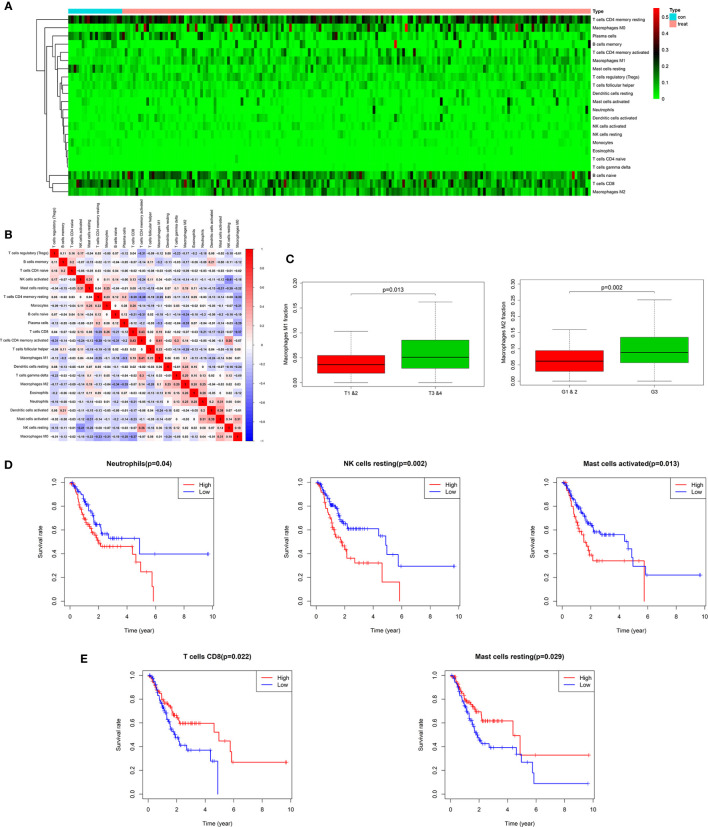
Twenty-two subpopulations of immune infiltration cells in AEG tissues. Heat map of the 22 immune cells proportions **(A)**. Correlation of CD4^+^ memory activated T cells, CD8^+^ T cells, M1 macrophages with other immune cells in TCGA **(B)**. M1 and M2 macrophages were obviously related to stage and histopathologic grading in patients with AEG (p < 0.05). Fraction of M2 macrophages were higher in G3 than in G1&2 tumors. M1 macrophages were lower in T1&2 tumors than in T3&4 tumors **(C)**. Kaplan-Meier curve for prognostic model showed that high overall survival rates related with fraction of the specific immune cell populations. The high fraction of neutrophils, resting NK cells, and activated mast cells were risk factors to predict survival rate in TCGA cohort **(D)**. The high fraction of CD8^+^T cells and resting mast cells were protective factors to predict survival rate in TCGA cohort **(E)**.

Differential infiltration by the 22 immune cell types identified in TCGA cohort was evaluated using the Wilcoxon signed rank test. For conducting survival analysis, the relationship between the inferred percentage of immune cells and survival has been illustrated using **Figures 1D** and **E**. Kaplan-Meier curves revealed correlations involving immune cell infiltrates and disease-free survival. All analyses were conducted using R v.3.5.2. The probability of differences between HD/AEG patients was assessed using the Student’s *t*-test (2-tailed Mann-Whitney U-test). Data are presented as mean ± SD. Spearman correlation analysis was used to analyze potential correlations involving the two datasets. The level of statistical significance was set at p < 0.05. Statistical analyses were performed using the GraphPad Prism v.8.0 software (GraphPad Software, La Jolla, USA).

## Results

### The Landscape of Immune Cells Infiltrating AEG Tissues in TCGA Cohort

The landscape of AEG-infiltrating immune cells has not been fully elucidated. We first investigated immune infiltration of AEG tissues in 22 subpopulations of immune cells using the CIBERSORT algorithm. **Supplementary Figure 1A** illustrates the fractions of immune cells in each AEG sample with the use of different colors, and the lengths of the bars in the bar plot indicate the levels of immune cell fractions. The fraction of immune cells varied remarkably among the samples. No significant fractions of neutrophils or monocytes were observed in AEG tumor tissues and adjacent normal tissues. As shown in **Figure 1A**, using unsupervised hierarchical clustering, the levels of naïve B cells, CD8^+^ T cells, CD4^+^ memory resting T cells, plasma cells, M1 macrophages, and M0 and M2 macrophages were relatively high in the samples of AEG tissues. Studies regarding pancreatic cancer have shown that the degree of histologic response to therapy is related to the proportion of infiltrating CD8^+^T cells and MDSCs, and interaction involving immune cells may affect the rate of metastasis (30). As shown in **Figure 1B**, fractions of different immune cells were correlated in AEG tissues in TCGA cohort in a weak or moderate manner. Populations with a statistically significant negative correlation included CD4^+^ memory resting T cells and CD4^+^ memory activated T cells (−0.39); resting NK and activated NK cells (−0.41); M2 macrophages and CD8^+^ T cells (−0.37); M0 macrophages and CD4 memory activated T cells (−0.37); and M0 macrophages and CD8^+^ T cells (−0.37). Populations with a statistically significant positive correlation included CD8^+^ T cells and CD4^+^ memory activated T cells (0.43); CD4^+^ memory activated T cells and M1 macrophages (0.41); CD4^+^ memory activated T cells and resting NK cells (0.36); activated mast cells and activated DCs (0.39); and resting NK cells and T-follicular helper cells (0.36). Overall, these results indicated that heterogeneity in terms of immune cell infiltration and interactions involving immune cells in AEG might have clinical significance.

### Clinical Characteristics and Immune Cells in AEG

In this study, we downloaded clinical AEG datasets from TCGA database, which included data based on certain clinical features, such as age, gender, histopathologic grading (G), American Joint Committee on Cancer (AJCC)/Union for International Cancer Control (UICC) stages, survival rate, primary tumor (T) stage, and lymph node stage (N). By comparing these clinical features, we found that the proportions of several immune cell types were significantly related to histopathological grading and primary tumor staging. However, there was no evident relationship with age, gender, or lymph node stage (**Supplementary Figure 1B**). After further analyses, we found that the M2 macrophage proportions were higher in G3 tumors than those in G1 and G2 tumors (p < 0.05) (**Figure 1C**). M1 macrophages were more abundant in T3 and T4 tumors than those in T1 and T2 tumors (p < 0.05) (**Figure 1C**). Classical M1 macrophages possess the ability to promote inflammation and activate the immune system. However, M2 macrophage activation involves anti-inflammatory and pro-tumoral effects realized through the establishment of critical interactions with cells related to tumor progression, such as MDSCs. This indicates that MDSCs and M2 macrophages may function in concert to resist the anti-tumor effects of M1 macrophages and promote the progression of AEG.

### Correlations Between Infiltrating Immune Cells and Overall Survival in AEG

Our study showed that overall survival was partly reflected by discrepancies in immune cell infiltration levels among patients with AEG. Kaplan-Meier curve analysis of the above-mentioned immune cells is depicted in **Figures 1D** and **E**. Neutrophils, resting NK cells, and activated mast cells were related to poor prognosis of patients with AEG (**Figure 1D**). In contrast, CD8^+^T cells and resting mast cells were associated with a good prognosis in patients with AEG (**Figure 1E**). These findings indicated that different types of infiltrating immune cells could result in different survival rates.

### Patient Characteristics Considered for MDSC Analysis

As described previously, MDSCs share the same cellular origins as neutrophils and macrophages with pro-tumor effects. Furthermore, according to the results of the CIBERSORT algorithm, neutrophils and macrophages are both important regulators in the TME. Subsequently, with an aim to provide a full description of infiltrating inflammatory cells, we examined MDSCs and their subtypes in AEG patients at our institute. All patients with a median age of 64 years (46–81 years) were pathologically diagnosed with AEG. Of the 46 patients selected, 39 (85%) were male and 7 were female (15%). Twelve patients (26%) received neoadjuvant chemotherapy. According to the guidelines prescribed by the eighth edition of the AJCC/UICC Staging Manual and Handbook, our study included 7 patients at stage I, 4 patients at stage II, 19 patients at stage III, and 16 patients with stage IV. Most patients (72%) presented with AEG histology only, and the remaining cases (28%) presented with mixed histology that included ring cell carcinoma or mucinous adenocarcinoma components (**Table 1**). And **Supplementary Table 1** showed the type of chemotherapy given to those patients who received neoadjuvant therapy.

**Table 1:** Patients’ Clinicopathological Characteristics.

Factor	N% or median (range)
Sex	
Male	39 (85%)
Female	7 (15%)
Age at diagnosis	64 (46-81)
Smoking history	
Never	21 (46%)
Former	11 (24%)
Current	14 (30%)
Neoadjuvant	
Yes	12 (26%)
No	34 (74 %)
Histology	
Mixed	13 (28%)
Pure	33 (72%)
Path T-stage	
Tis	1 (2%)
T1	2 (4%)
T2	6 (13%)
T3	8 (17%)
T4	29 (64%)
Path N-stage	
N0	19 (41%)
N1	13 (28%)
N2	10 (22%)
N3	4 (9%)
Clinical stage	
I	7 (15%)
II	4 (9%)
III	19 (41%)
IV	16 (35%)

### A Population of MDSCs and Three Subsets in PB and HD

**Figure 2:**
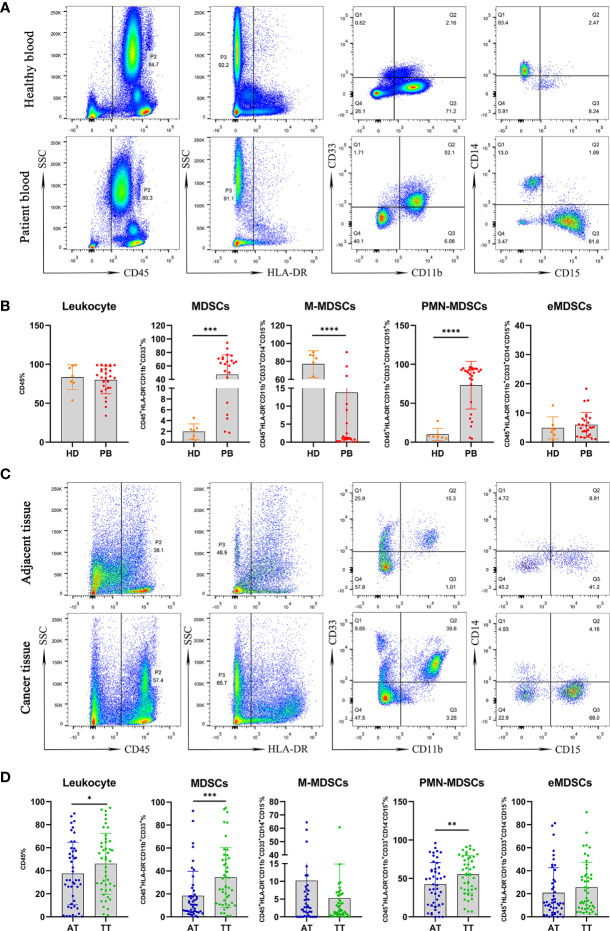
Mononuclear cells obtained from peripheral blood of AEG patients (n =27) and HD (n = 7) were assessed by multicolor flow cytometry. Mononuclear cells were stained for MDSCs using fluorochrome-labeled antibodies against CD45, HLA-DR, CD11b, CD33, CD14, CD15. A morphological gate of mononuclear cells based on SSC and SFC properties were applied before gating for MDSCs subsets. Markers analyzed are indicated in the axis of each flow cytometry graphs. Representative dot plots of leukocyte (CD45^+^cells), MDSCs (CD45^+^HLA-DR^−^CD11b^+^CD33^+^cells) and three subsets of MDSCs, including monocytic (M)-MDSCs (CD45^+^HLA-DR^−^CD11b^+^CD33^+^CD14^+^CD15^−^ cells), polymorphonuclear (PMN)-MDSCs (CD45^+^HLA-DR^−^CD11b^+^CD33^+^CD14^−^CD15^+^ cells), and early-stage eMDSCs (CD45^+^HLA-DR^−^CD11b^+^CD33^+^CD14^−^CD15^−^ cells) identified using a gating strategy were shown **(A)**. The frequencies of leukocyte, MDSCs, M-MDSCs, PMN-MDSCs, and eMDSCs in the PB and HD are presented as the percentage within the total respective subset **(B)**. Comparative analysis of levels of leukocyte and MDSCs in AT (n = 46) and TT (n = 46) from AEG patients. Representative flow cytometric plots show leukocyte, MDSCs, and three subsets of MDSCs in AT and TT of AEG patients **(C)**. Cumulative scatter plots show the percentage of leukocyte, MDSCs, M-MDSCs, PMN-MDSCs, and eMDSCs in the AT and TT, which are presented as the frequency within the total respective subset **(D)**. Each point corresponds to an individual patient or a HD. Column graphs show the mean ± standard error of the mean (SEM). Asterisks represent statistical significance (*p < 0.05; **p < 0.01; ***p < 0.001; ****p < 0.0001).

**Figure 2A** shows the gating strategy used to analyze MDSCs and three subsets in peripheral blood. There were no differences in the level of leukocytes in peripheral blood between PB and HD groups. However, the frequency of MDSCs and PMN-MDSCs in peripheral blood was higher in PB than that in HD (47.15%/73.24% *vs.* 1.947%/10.03%, p < 0.001, p < 0.0001, respectively). In contrast, the abundance of M-MDSCs was greater in the HD group than that in the PB cohort (77.09 *vs.* 13.81%, p < 0.0001). However, there were no significant differences in the levels of early-stage MDSCs (eMDSCs) between the PB and HD groups (**Figure 2B**).

We used the same gating strategy to analyze the population of MDSCs and their three subsets in AT and TT from AEG patients (**Figure 2C**). The frequencies of leukocytes, MDSCs, and PMN-MDSCs in peripheral blood were higher in TT than those in AT (46.06%/34.45%/55.30% *vs.* 37.53%/18.39%/42.26%, p < 0.05, p < 0.001, p < 0.01, respectively). However, the levels of M-MDSCs and eMDSCs were not significantly different between the TT and AT groups (**Figure 2D**).

**Figure 3:**
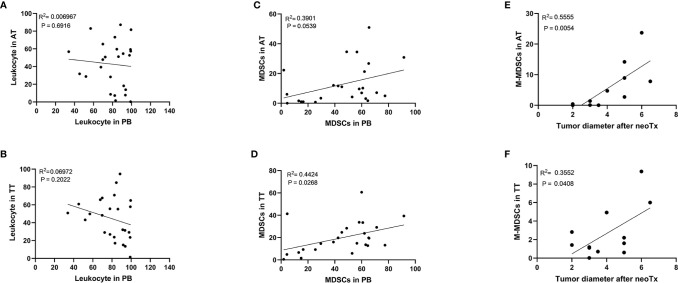
There was no significant correlation between the frequency of the leukocyte in PB and the frequency of the leukocyte in AT **(A)** or TT **(B)**. Positive correlation between the frequency of the MDSCs in PB and the frequency of the MDSCs in AT **(C)** or TT **(D)**. The positive correlation between the tumor diameter after neoTx and the frequency of the M-MDSCs in AT **(E)** or TT **(F)** was determined. Pearson correlation coefficient (R^2^) and statistical significance are presented for each correlation.

Furthermore, no correlation was observed between the levels of leukocytes in AT/TT and PB (**Figures 3A, B**). This showed that the level of leukocytes in PB did not reflect the degree of leukocyte infiltration in tissues. However, we observed a positive correlation between the level of MDSCs in AT/TT and PB, especially TT and PB, using Pearson correlation analysis (R^2^ = 0.3901, p = 0.0539; R^2^ = 0.4424, p = 0.0266, respectively; **Figures 3C, D**). Furthermore, it was observed that the tumor diameter after neoadjuvant therapy (neoTx) and the level of M-MDSCs in AT/TT exhibited evident positive relationships (R^2^ = 0.5555, p = 0.0054; R^2^ = 0.3552, p = 0.0408, respectively; **Figures 3E, F**). Thus, it could be implied that the level of M-MDSCs in AEG tumor tissues might be considered an important early indicator for predicting clinical responses to chemotherapy.

### Clinical Significance of MDSCs

**Figure 4:**
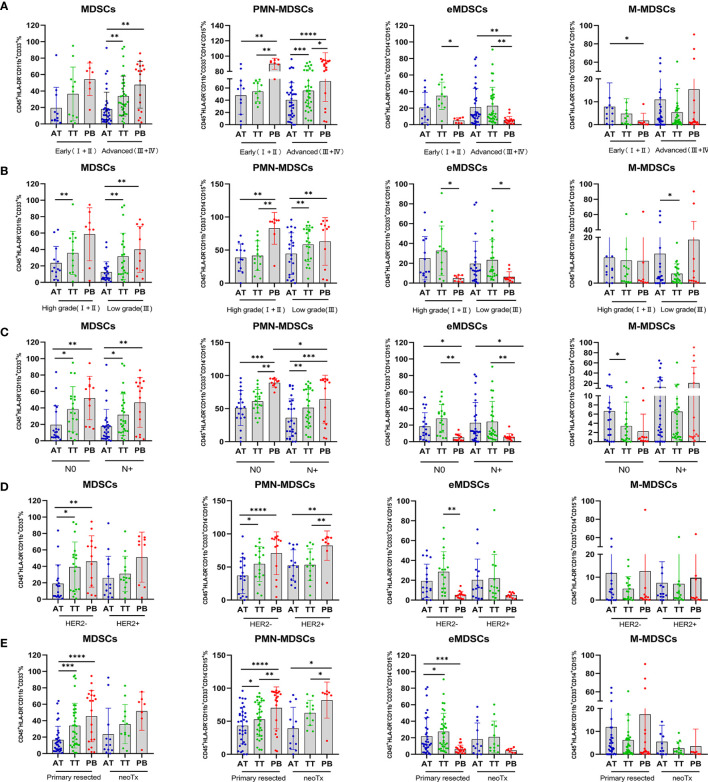
Percentage of MDSCs and three subsets of MDSCs in AEG patients with different clinicopathological characteristics. Analysis of MDSCs, PMN-MDSCs, eMDSCs, and M-MDSCs, cells frequencies in adjacent tissues (AT), tumor tissues (TT), and peripheral blood (PB) of AEG patients at different staging (early ;+ *vs.* advanced +) **(A)**, histopathologic grading (low *vs.* high) **(B)**, lymph node metastasis (N0 *vs.* N+) **(C)**, HER2 expression (HER2− vs. HER2+) **(D),** and therapy (primary resected *vs.* neoTx) **(E)** were examined by multicolor flow cytometry. The frequencies of MDSCs, PMN-MDSCs, eMDSCs, and M-MDSCs in the AT (n = 46), TT (n = 46), and PB (n = 27) are presented as the percentage of the total respective subset. The percentages of MDSCs subsets from the three tumor microenvironments were determined in the same patients as described in Figure 2. Each point corresponds to an individual AEG patient. Data depict the mean ± SEM. The p values are represented as follows: (*p < 0.05; **p < 0.01; ***p < 0.001; ****p < 0.0001).

To explore the clinical significance of MDSC subsets, we analyzed their relationship with patient clinicopathological characteristics. Interestingly, tumor progression analysis revealed higher levels of PB-infiltrating MDSCs in AEG patients with advanced *vs.* early-stage disease, HER 2^−^ vs. HER2^+^, and primary resected malignancies *vs.* neoTx (p < 0.01, p < 0.01, p < 0.0001, respectively; **Figures 4A, D, E**). Thus, circulating MDSCs may be considered potential markers for tumor progression in cancer therapy. Although the abundance of PB-infiltrating eMDSCs exhibited the opposite trend, lower levels of PB-infiltrating eMDSCs were observed in these patients (p < 0.01, p < 0.01, p < 0.001, respectively; **Figures 4A, D, E**). Moreover, the predominance of PMN-MDSC discrepancy was mainly attributed to the infiltration of tissues, and there was no evident difference in AEG patients with different clinical stages (**Figures 4A, D, E**). We observed elevated levels of PMN-MDSCs in the TT *vs.* AT from advanced stage, low grade, lymph node metastasis, HER2^−^, and primary resected AEG patients (p < 0.001, p < 0.01, p < 0.01, p < 0.05, p < 0.05, respectively; **Figures 4A–E**). We also observed higher levels of AT-infiltrating M-MDSCs in AEG patients presenting with early *vs.* advanced tumor stage, low *vs.* high grade, and N0 *vs.* N+ (p < 0.05, p < 0.05, p < 0.05, respectively; **Figures 4A–C**). These results indicated that infiltrating PMN-MDSCs in TT played a major role in tumor progression, whereas M-MDSCs mainly infiltrated AT without penetrating the tumor.

### Activation of the IL-6/STAT3 Pathway in MDSCs

**Figure 5:**
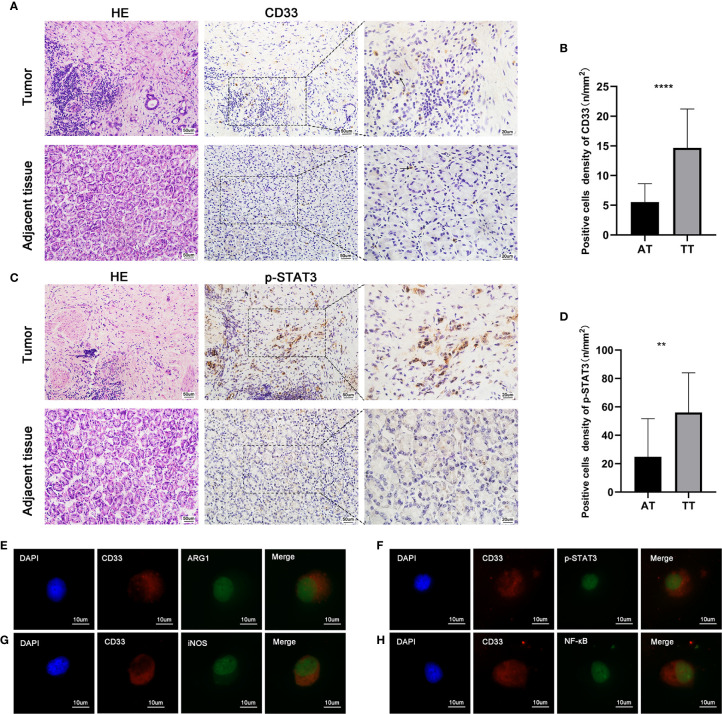
The paraffin-embedded AEG tissues were collected for p-STAT3 and CD33 staining. Histopathological appearance of AEG tumor tissues and adjacent non-tumorous tissues and immunohistochemical representative images of CD33+ (MDSCs) **(A)**, p-STAT3**(C)** in tumor tissue and adjacent non-tumorous tissues. There was weak or negative expression of p-STAT3 in adjacent normal tissues. However, there was strong expression of p-STAT3 in AEG tissues mainly localized in the nuclei of tumor epithelial cells and stroma cells. Quantitative analysis of CD33 **(B)** and p-STAT3 **(D)** positive staining in AEG and adjacent normal tissues. Representative double immunofluorescence pictures of the co-localization of CD33 (red) and AEG1 (green) **(E)**. Representative double immunofluorescence pictures of the co-localization of CD33 (red) and p-STAT3 (green) **(F)**. Representative double immunofluorescence pictures of the co-localization of CD33 (red) and iNOS (green) **(G)**. Representative double immunofluorescence pictures of the co-localization of CD33 (red) and NF-κB (green) **(H)**. Data depict the mean ± SEM. The p values are represented as follows: (**p < 0.01; ****p < 0.0001). Black arrows showed CD33^+^ cells. 20× magnification, scale bar = 50 um; 40× magnification, scale bar = 20 um; 100× magnification, scale bar = 10 um, applicable to all panels.

Increasing evidence indicates that the IL-6/STAT3 pathway is associated with the functional properties of cells that form the TME (31–33). Based on the reported immunosuppressive potential of MDSCs in malignant tumors, we analyzed the levels of key immunosuppressive mediators (IDO, ARG1, IL-6, and TGF-β1) in AEG patients. These cytokines play vital roles in the generation and expansion of MDSCs during tumorigenesis. Compared with HD, we observed higher levels of IL-6 in PB; however, no statistically significant difference was observed. Additionally, the levels of IDO, TGF-β1, and ARG1 were not significantly different between HD and PB (**Supplementary Figure 1C**). CD15^+^ cells were isolated from AEG tumor tissues using an immunomagnetic cell sorting method (MACS). Double immunofluorescence staining of cytocentrifuge smears revealed co-localization, indicating that MDSCs expressed ARG1, iNOS, and NF-κB, which have been reported to participate in suppressor T cell functions (**Figures 5E–G**) (34).

It has been recognized that activation of p-STAT3 is a key factor in cells that constitute tumor stroma and lead to cancer pathogenesis. Abnormal activation of STAT3 in cancer is associated with the quantity and function of immunosuppressive tumor-promoting MDSCs (28). In our study, immunohistochemistry was used to detect the intensity, distribution, and number of MDSCs (**Figure 5A**), as well as the activation status of p-STAT3 (**Figure 5C**). CD33 protein expression, which is a representative marker of MDSCs, was analyzed by performing quantitative immunohistochemistry. The number of CD33^+^ cells was also higher in AEG tissues than that in adjacent tissues (p < 0.05) (**Figure 5B**). Activation and expression of p-STAT3 was weak in adjacent tissues. However, strong expression of p-STAT3 was observed in AEG tissues (**Figure 5D**). Double immunofluorescence labeling also showed co-localization of CD33 and p-STAT3, which indicated activation of the STAT3 signaling pathway in MDSCs (**Figure 5H**). Collectively, we observed that p-STAT3 expression in tumor stroma cells was a critical contributor to cancer pathogenesis. *In vivo*, targeting of the STAT3 signaling pathway in tumor-associated MDSCs leads to the elicitation of enhanced CD8^+^ T cell responses, activation of tumor-associated monocytes and DCs, and tumor regression (35).

### Characterization of Immune Cells Infiltrating AEG Tumors

**Figure 6:**
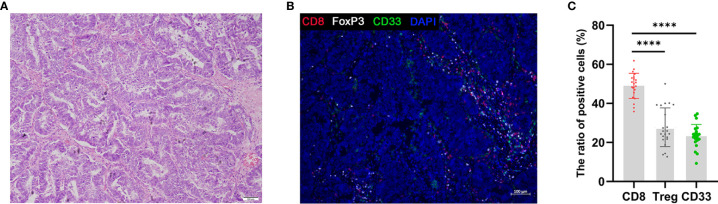
Triple fluorescent immunohistochemistry of AEG tumors shows T-cell infiltration. H&E staining of AEG tumor tissue **(A)**. Representative image after multispectral imaging for CD8, FoxP3, CD33 together with a nuclear marker, DAPI **(B)**. Tumor sections were sequentially stained using Opal fluorophores for CD8 (red), FoxP3 (white), CD33 (green), and DAPI (blue). The ratio of positive cells in immune cells was defined as the ratio of the number of each positive immune cells to the whole number of each immune cells (red, white, and green) in a 4-mm^2^ field of view **(C)**. Data depict the mean ± SEM. The p values are represented as follows: (****p < 0.0001) **(C)**. 10× magnification, scale bar = 100 um, applicable to all panels.

Developing tumors recruit diverse immune cells that constitute the TME, and through the establishment of iterative interactions, both the tumor cells and their microenvironment undergo coevolution (36). Previously, during the investigation of tumor progression, CTLs were considered as favorable prognostic factors; however, presently, immunosuppressive cells, such as FOXP3^+^ Tregs and MDSCs, are considered as unfavorable factors affecting patient prognosis (37). Accordingly, we investigated infiltration of the AEG TME by immune cells using triple immunohistochemical staining (**Figure 6B**) and quantification (**Figure 6C**) experiments. The presence of AEG tumors was confirmed by performing H&E staining (**Figure 6A**). Triple immunofluorescence staining results showed that CD8^+^ T cells, Tregs, and MDSCs were located primarily at the AEG tumor-invasive edge adjacent to the tumor stroma (**Figure 6B**). Our data indicated that AEG tumors exhibited an inflamed tumor phenotype, with relatively more infiltrating CD8^+^ T cells and relatively fewer Treg cells and MDSCs. Thus, the proportion of infiltrating CD8^+^ T cells and MDSCs and the proportion of infiltrating CD8^+^ T cells and Tregs related to the degree of histologic responses to therapy and prognosis of AEG patients warrant further exploration and validation.

## Discussion

In recent years, the rate of adenocarcinoma incidence involving the AEG has increased worldwide. The use of immune checkpoint inhibitors (ICIs), such as anti-PD-1, anti-CTLA4, and anti-PD-L1 antibodies, has demonstrated significant benefits in various cancers, including gastric adenocarcinomas (38, 39). However, responses to therapy vary widely among patients. The low response rates of ICI are attributed to the complex TME, which is a milieu consisting of tumor cells, stromal cells, and immune cells (40). Several studies have shown that tumor-infiltrating immune cell subsets, such as CD8^+^ T cells, DCs, and memory T cells, are increased in cancer patients and usually associated with a good prognosis, while immunosuppressive cell populations, regulatory T cells, and MDSCs exhibit the opposite trend (9, 41, 42). Owing to technical limitations, most of the published studies used immunohistochemistry based on single cell-surface markers to assess the extent of tumor infiltration by immune cells. However, this method is prone to bias in distinguishing cell types with closely related phenotypes. Therefore, inconsistent results have often been observed in clinical studies. Using the CIBERSORT algorithm, it is now possible to accurately analyze the relative proportions of various immune cell subpopulations and to overcome the disadvantages/inaccuracies associated with traditional immunohistochemistry (43).

In our study, the CIBERSOFT algorithm was applied to explore differences between immune cells infiltrating AEG tissues and adjacent normal tissues. We found a relatively high proportion of naïve B cells, CD8^+^ T cells, CD4^+^ memory resting T cells, plasma cells, and M1, M0, and M2 macrophages infiltrating AEG tissue samples. However, the TME is a complex milieu, and the relationships among various immune cells are diverse. Many previous studies have indicated that CD8^+^ T cells, CD4^+^ memory activated T cells, NK cells, T-follicular helper cells, and activated DCs are protective factors in tumor patients (9). We observed statistically significant positive correlations involving CD8^+^ T cells and CD4^+^ memory activated T cells (0.43); CD4^+^ memory activated T cells and M1 macrophages (0.41); CD4^+^ memory activated T cells and resting NK cells (0.36); resting NK cells and T-follicular helper cells (0.36); and mast cell activation and DC activation (0.39) in AEG patients. Activated mast cells are critical factors in the regulation of Treg immune suppression (44). Therefore, the observed correlation between activated mast cells and activated DCs warrants further research. In contrast, M2 macrophages are an unfavorable factor in terms of promoting tumor growth, and we observed negative correlations between M2 macrophages and CD8^+^ T cells (−0.37); M0 macrophages and CD4 memory activated T cells (−0.37); and M0 macrophages and CD8^+^ T cells (−0.37). M0 macrophages can be transformed into M1 (anti-tumor phenotype) or M2 (tumor-promoting phenotype) cells. Consequently, the correlation between M0 macrophages and CD4 memory activated T cells and CD8^+^ T cells also warrants further research, and we examined potential correlations involving immune cells and clinical characteristics. Although neutrophils were not significantly correlated with pathologic stage (p > 0.05), a high neutrophil fraction was observed and determined as a risk factor and was correlated with poorer patient outcomes.

MDSCs are a critical component of the TME, and share a similar origin to neutrophils and macrophages. Substantial evidence has implied that MDSCs play important roles in tumor progression, drug resistance, and tumor immune escape (45–47). Data on the relative gene expression by MDSCs were not included in TCGA database. Therefore, to provide a complete landscape of the TME, we detected the levels of leukocytes and MDSCs infiltrating AEG by performing flow cytometry and analyzed correlations between MDSCs and clinical characteristics. Compared with the CIBERSOFT algorithm, flow cytometry analysis could directly reflect the components of MDSCs, which helped to detect differences in the levels of leukocytes and MDSCs in the peripheral blood and specimens of the AEG patients and healthy donors. Our results showed that the levels of leukocytes in the peripheral blood of patients and HD were similar; however, PB mainly comprised PMN-MDSCs, and HD mainly consisted of M-MDSCs. Paired AEG tumor tissues and adjacent tissues were mainly infiltrated by PMN-MDSCs, and the relatively high level of leukocytes in tumor tissues might be attributable to the abundant blood supply surrounding tumor tissues. Correlation analysis showed that the levels of MDSCs in PB were significantly correlated with the levels of MDSCs in tumor tissues, but no evident correlation involving adjacent tissues was observed. In AEG patients with different clinicopathological characteristics, the infiltration levels by MDSCs and their subtypes were significantly different, indicating that the discrepancy in the abundance of MDSCs in different tissues and the immunosuppressive function of MDSCs subtypes differed, and this resulted in the differences in patient prognosis.

The JAK-STAT pathway is an important oncogenic signaling cascade that is strictly regulated under physiological conditions. However, in most malignant tumors, STAT3 is abnormally activated *via* tyrosine phosphorylation (48). The most common mechanism is through increased/sustained IL- 6 (family)/gp130 signal transduction, and aberrant signaling by EGFR, HER2, Ras, and other oncogenic pathways leads to increased IL-6 production and STAT3 activation (49, 50). Excessive STAT3 activation, in turn, enhances the number and activity of MDSCs and prevents the differentiation and expansion of functional DCs (35). In mouse models of hepatocellular carcinoma and melanoma, reductions in tumor burden are related to diminished IL-6 levels and impaired MDSC function (34, 51).

The STAT3 and NF-κB pathways have been confirmed to play roles in inflammatory signaling cascades. STAT3 increases NF-κB activity, and persistent activation of STAT3 is dependent on NF-κB signaling (52). Double immunofluorescence staining revealed the activation of STAT3 and NF-κB pathways in MDSCs, which were associated with AEG progression and immune tolerance. MDSCs express high levels of both ARG1 and iNOS, and exhibit increased production of reactive oxygen species (ROS) to mediate T-cell suppression. Double immunofluorescence labeling results indicated that MDSCs could express substantial levels of ARG1 and iNOS, which was consistent with the findings of a previous report (53).

During cancer progression, CTLs undergo dysfunction and exhaustion due to immune-related tolerance and immunosuppression in the TME, and this favors the development of adaptive immune resistance (8). MDSCs, M2 macrophages, and Tregs can result in the formation of immunologic barriers against CD8^+^ T cell-mediated anti-tumor immune responses (54). Therefore, CD8^+^ T cells should be activated into effector CTLs in the process of tumor immunity to generate durable and efficient antitumor immune responses. Consistent with previous reports, we observed relatively higher extent of CD8^+^ T cell infiltration in AEG tumors (55). However, as discussed above, single cell types have poor predictive power. Therefore, whether the proportion of infiltrating CD8^+^ T cells and MDSCs, and the proportion of infiltrating CD8^+^ T cells and Tregs is related to the degree of histologic response to therapy and the prognosis of AEG patients should be further explored.

## Data Availability Statement

The original contributions presented in the study are included in the article/**Supplementary Material**. Further inquiries can be directed to the corresponding authors.

## Ethics Statement

The studies involving human participants were reviewed and approved by the Ethics Committee of Tianjin Medical University in Tianjin Medical University Cancer Institute and Hospital. The patients/participants provided their written informed consent to participate in this study.

## Author Contributions 

YW, HS, and LG: design and initiation of the study, quality control of data, data analysis and interpretation, and manuscript preparation and editing. NZ, XW, and ZS: data acquisition. ZY: study concept and design and initiation of the study. All authors contributed to the article and approved the submitted version.

## Funding

This study was supported by the National Natural Science Foundation of China (Grant Numbers: 81772619 and 81501994), Clinical Trial Project of Tianjin Medical University (Grant Number: 2017kylc008), Tianjin Medical University Cancer Institute and Hospital Clinical Trials (C1711), Wu Jieping Medical Foundation (Grant Number: 320.2730.1886), and Bethune Charity Foundation (Grant Number: HZB-20190528-18 and HZB-20190528-11).

## Conflict of Interest

The authors declare that the research was conducted in the absence of any commercial or financial relationships that could be construed as a potential conflict of interest.

## Supplementary Material

Supplementary Figure 1 Analysis of immune cells proportions in 422 tumor and 39 normal samples in TCGA by using the CIBERSORT algorithm **(A)**. These immune cells were obviously related to stage and histopathologic grading in patients with AEG (P < 0.05) **(B)**. MDSCs related immunosuppressive factors, ARG1 activity, the concentration of indoleamine 2,3-dioxygenase (IDO), interleukin 10 (IL-10), and transforming growth factor β1 in plasma of AEG patients and HD were analyzed. Each point corresponds to a single patient or HD **(C)**.
