# Supplementary figures and images for: Myeloid-Derived Suppressor Cells in Immune Microenvironment Promote Progression of Esophagogastric Junction Adenocarcinoma
Source: Front Oncol. 2021 Mar 29;11:640080. doi: 10.3389/fonc.2021.640080 (PMC8039399; doi:10.3389/fonc.2021.640080)

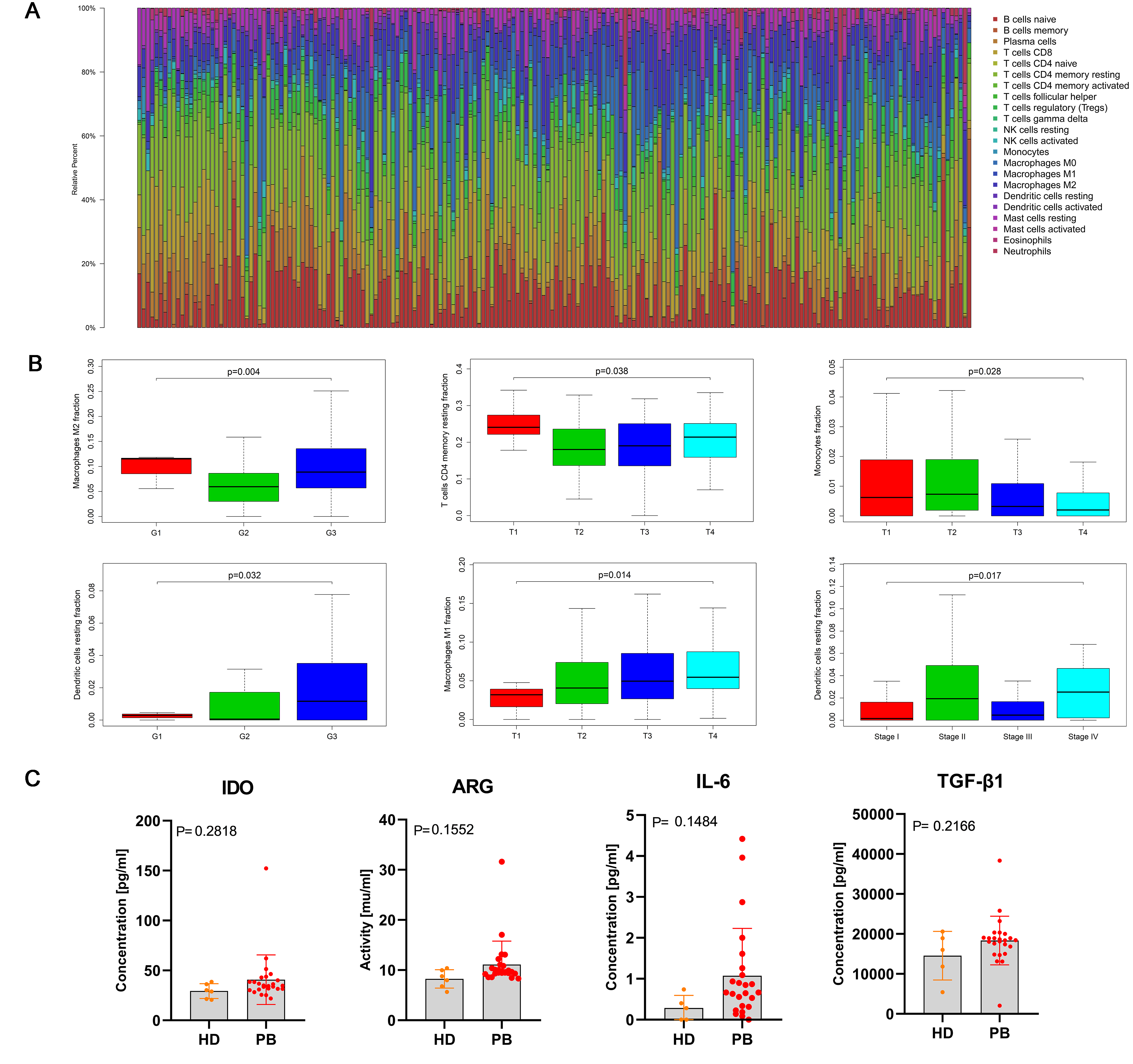

Supplement: Supplementary Figure 1 — Analysis of immune cells proportions in 422 tumor and 39 normal samples in TCGA by using the CIBERSORT algorithm (A). These immune cells were obviously related to stage and histopathologic grading in patients with AEG (P < 0.05) (B). MDSCs related immunosuppressive factors, ARG1 activity, the concentration of indoleamine 2,3-dioxygenase (IDO), interleukin 10 (IL-10), and transforming growth factor β1 in plasma of AEG patients and HD were analyzed. Each point corresponds to a single patient or HD (C). [file Image_1.tif]
