# Supplementary material for: Myeloid-Derived Suppressor Cells in Immune Microenvironment Promote Progression of Esophagogastric Junction Adenocarcinoma
Source: Front Oncol. 2021 Mar 29;11:640080. doi: 10.3389/fonc.2021.640080 (PMC8039399; doi:10.3389/fonc.2021.640080)
Supplement: Supplementary file 2 [file Table_1.docx]

| **Type of chemotherapy** | **Patients** **received neoadjuvant**  **chemotherapy (N = 12)** |
| --- | --- |
|  | **N** |
| docetaxel + floxuridine + cisplatin | 4 |
| oxaliplatin + tegafur | 2 |
| lapatinib + capecitabine + oxaliplatin | 1 |
| docetaxel + oxaliplatin + tegafur + calcium folinate | 1 |
| cisplatin + docetaxel + tegafur | 1 |
| oxaliplatin + S-1 | 1 |
| docetaxel + nedaplatin + tegafur | 1 |
| oxaliplatin + tetrahydrofolate + 5‐FU | 1 |

Supplementary Table1 Type of neoadjuvant therapy received by the patients
